# Differences in Glycerolipid Response of *Chlamydomonas reinhardtii* Starchless Mutant to High Light and Nitrogen Deprivation Stress Under Three Carbon Supply Regimes

**DOI:** 10.3389/fpls.2022.860966

**Published:** 2022-05-06

**Authors:** Miao Yang, Xi Xie, Fan-Tao Kong, Kun-Peng Xie, Si-Hui Yu, Jing-Yi Ma, Song Xue, Zheng Gong

**Affiliations:** ^1^Key Laboratory of Plant Biotechnology of Liaoning Province, School of Life Sciences, Liaoning Normal University, Dalian, China; ^2^Dalian Key Laboratory of Marine Bioactive Polypeptide Drugs, School of Life Sciences, Liaoning Normal University, Dalian, China; ^3^Liaoning Ocean and Fisheries Science Research Institute, Dalian, China; ^4^School of Bioengineering, Dalian University of Technology, Dalian, China

**Keywords:** *Chlamydomonas*, starchless mutant, autotrophic, mixotrophic, triacylglycerol accumulation, membrane lipid

## Abstract

Carbon source serves as a crucial factor for microalgal lipid biosynthesis. The supplied exogenous inorganic or organic carbon affects lipid accumulation in microalgae under stress conditions. However, the impacts of different carbon availability on glycerolipid metabolism, triacylglycerol (TAG) metabolism in particular, still remain elusive in microalgae. *Chlamydomonas* starchless mutant BAFJ5 has emerged as a model system to study TAG metabolism, due to its property of hyper-accumulating TAG. In this study, the glycerolipidomic response of the starchless BAFJ5 to high light and nitrogen-deprived (HL-N) stress was deciphered in detail to distinguish glycerolipid metabolism under three carbon supply regimes. The results revealed that the autotrophically and mixotrophically grown BAFJ5 cells aerated with air containing 2% CO_2_ presented similar changes in growth, photosynthetic activity, biochemical components, and glycerolipid metabolism under HL-N conditions. But the mixotrophically grown BAFJ5 aerated with air containing 0.04% CO_2_ exhibited more superior accumulation in TAG, which was esterified with a significantly higher proportion of C18:1n9 and prominently the lower proportions of polyunsaturated fatty acids. In addition, these cells increased the relative levels of C18:2n6 in the membrane lipids, i.e., monogalactosyldiacylglycerol (MGDG) and digalactosyldiacylglycerol (DGDG), in priority, and decreased that of C18:3n3 and C18:4n3 in the betaine lipid, *N*,*N*,*N*-trimethylhomoserine diacylglycerol (DGTS), subsequently, to adapt to the HL-N stress conditions, compared to the cells under the other two conditions. Thus, it was suggested that *C*. *reinhardtii* starchless mutant appeared to present distinct metabolism for TAG biosynthesis involving membrane lipid remodeling under distinct carbon supply regimes. This study provides insights into how the different carbon supply regimes affect lipid metabolism in Chlamydomonas starchless cells, which will benefit the optimized production of storage lipids in microalgae.

## Introduction

Microalgae have received intensive attention as feedstocks for the production of renewable energy, such as biofuels, due to their ability to produce large amounts of energy-rich triacylglycerol (TAG) under adverse environmental conditions (Hu et al., 2008). Nitrogen starvation has emerged as an effective strategy to enhance TAG accumulation in microalgae (Zienkiewicz et al., 2016). In many microalgae, in particular green algae, the photosynthetically assimilated carbon could be partitioned into the storage molecules, including starch and TAG, though the interaction between starch and TAG biosynthesis still remains poorly understood (Pick and Avidan, 2017). These studies have demonstrated that shunting carbon precursors from starch formation lead to hyper-accumulation of fatty acids and thus TAG, in green microalgae, e.g., *Chlamydomonas reinhardtii* (Li et al., 2010a,b), *Scenedesmus obliquus* (Breuer et al., 2014; de Jaeger et al., 2014), and *Chlorella sorokiniana* (Vonlanthen et al., 2015; Wu et al., 2019), under nitrogen deprivation.

Apart from stress induction together with blocking the competitive starch biosynthesis, carbon availability has been shown to be another essential factor for TAG accumulation in green microalgae (Smith and Gilmour, 2018). Microalgae can grow autotrophically or mixotrophically, under distinct carbon sources, including inorganic carbon and organic carbon. The autotrophy is usually supplied with distinct ratios of CO_2_, and the mixotrophy is supplied with distinct ratios of CO_2_ and also organic carbon, e.g., acetate, glucose, etc. Organotrophic culture in PBRs also needs mixing with aeration, which contains 0.04% CO_2_. Additionally, the mixotrophic culture in flasks also contains 0.04% CO_2_ from the air. To our knowledge, substantial amounts of studies on lipid production in microalgae under either carbon supply condition have been widely reported (Gardner et al., 2013; Moon et al., 2013; Yang et al., 2020). However, the potential differences in lipid production among these distinct carbon supply conditions in stress-induced microalgae still remain largely unclear, TAG accumulation in particular. The mixotrophically grown microalgae usually provide more superior biomass production than those from photoautotrophic cultures under both favorable (Kim et al., 2013; Yun et al., 2021) and unfavorable (Shen et al., 2018, 2019; Smith and Gilmour, 2018) conditions. Previous studies showed that the nitrogen-starved microalgae, e.g., *C*. *vulgaris* (Shen et al., 2019), *S*. *obliquus* (Shen et al., 2018), and *C*. *reinhardtii* (Smith and Gilmour, 2018), presented more enhanced TAG accumulation under mixotrophic cultivation than that under autotrophic cultivation; in addition, the other biochemical components, such as protein and starch, also exhibited distinct differences between distinct trophic cultures. However, a majority of these studies were performed using the wild-type cells. *C*. *reinhardtii* starchless mutant BAFJ5, which is defective in ADP-glucose pyrophosphorylase, has emerged as a model system to study TAG metabolism, due to its property of hyper-accumulating TAG (Li et al., 2010a; Liu et al., 2016; Meng et al., 2020; Yang et al., 2020). Moreover, TAG accumulation was dramatically enhanced when exposed to high light in nitrogen-deprived BAFJ5 (Li et al., 2010a; Yang et al., 2020). Due to the defect of carbon partitioning to starch biosynthesis in BAFJ5, it makes easier to understand TAG metabolism involving membrane lipid remodeling. The wild-type *C*. *reinhardtii* strains were reported to accumulate minor TAG content (less than 2% of dry weight) (Li et al., 2010a; Siaut et al., 2011), making it certainly difficult to quantitatively monitor changes in fatty acid amounts and profiles of glycerolipids. In such a scenario, it might provide more opportunities to explore the subtle phenomenon using BAFJ5 as a model system, and the potentially distinct lipid remodeling and TAG biosynthesis were probably ignored using wild-type strain. Besides, the carbon flow of starchless mutant requires no excess shunt to another storage compound starch, more beneficial to understanding the turnover pathway concerning membrane lipids into TAG. For instance, we recently discovered that the galactolipid, digalactosyldiacylglycerol (DGDG), and betaine lipid, *N*,*N*,*N*-trimethylhomoserine diacylglycerol (DGTS), directed *de novo* synthesized linolenate into TAG under stress conditions using the BAFJ5 mutant as the research model (Yang et al., 2020). However, how do the stress-induced starchless mutants regulate the lipid metabolism, especially TAG biosynthesis, under distinct carbon supply regimes This question still remains to be elucidated.

In general, the inorganic carbon source under mixotrophic condition for *C*. *reinhardtii* includes the ambient 0.04% CO_2_ (Fan et al., 2011; Smith and Gilmour, 2018) or 2% CO_2_ (Meng et al., 2020; Yang et al., 2020). However, whether the lipid response under the two mixotrophic conditions exhibits similar or distinct features has still been unknown. In this work, three carbon supply regimes, i.e., mixotrophic 1 (17 mM acetate and 2% CO_2_, v/v), mixotrophic 2 (17 mM acetate and ambient 0.04% CO_2_, v/v), and autotrophic (2% CO_2_, v/v) culture modes, were conducted in photobioreactors (PBRs) under high light and nitrogen starvation (HL-N) conditions in starchless BAFJ5. To better understand TAG metabolism in the starchless mutant, its growth, photosynthetic activity, biochemical components, and glycerolipid response were monitored and further compared to distinguish the unique TAG characteristics under the three carbon supply regimes.

## Materials and Methods

### Microalgal Strains and Culture Conditions Under Three Carbon Supply Regimes

The Chlamydomonas starchless mutant, BAFJ5 (cw15 sta6, CC4348), from Chlamydomonas Resource Center^1^ was used and maintained in tris-acetate-phosphate (TAP) medium (Harris, 2009) under orbital shaking (80 rpm). The sub-culture was conducted under 12-h light/12-h dark cycle at 25°C, and the light intensity was set at 50 μmol photons m^–2^ s^–1^ (P9710, Gigahertz Optik, Germany). A two-stage culture, including the first stage of nitrogen repletion and the second stage of nitrogen depletion, was carried out to induce TAG accumulation in *C*. *reinhardtii* as previously described (Yang et al., 2017).

For nitrogen-replete cultivation, the algal cells were inoculated into a TAP medium with an initial optical density of 0.2 at 750 nm under 48 h of continuous illumination, and 50-μmol photons m^–2^ s^–1^ illumination was provided. The organic carbon in the form of acetate at 17.4 mM was added to the TAP medium. Glass air bubble column photobioreactors (50 mm in diameter, 450 mm in height, and 600 ml in culture volume; PBRs) were used to cultivate algal cells. These PBRs were bubbled with air (120 ml min^–1^) containing 2% CO_2_.

The harvested cells grown under nutrient-abundant conditions were first washed with TAP medium without nitrogen (TAP-N medium) and were diluted to cultures with an optical density of 1.0 at 750 nm in TAP-N medium. The three carbon supply regimes were applied to nitrogen-depleted cultivation for *C*. *reinhardtii* BAFJ5. The first regime, mixotrophic 1 mode, was a TAP-N medium supplied with air containing 2% CO_2_, the second regime, mixotrophic 2 mode, was TAP-N medium supplemented with air containing 0.04% CO_2_, and the third regime, autotrophic mode, was TAP-N medium without acetate and with air containing 2% CO_2_. The cultures were illuminated consecutively with an enhanced illumination of 500 μmol photons m^–2^ s^–1^ to promote TAG accumulation. The nitrogen-starved cultivation has proceeded in three batches, and each batch included one replicate for each culture mode. The stress treatment sustained 48 h and the algal cells were sampled at 0, 4, 12, 24, and 48 h, respectively. The samples were centrifuged at 4,000 rpm for 5 min and then lyophilized (LABCONCO 7740070, United States) for 4 h.

### Determination of Growth Parameters and Photosynthetic Activity

The growth parameters, including optical density at 750 nm (OD_750_) and cellular dry weight density (DW, mg mL^–1^), and the PS II quantum yield, i.e., *F*_*v*_/*F*_*m*_, were monitored as previously described (Yang et al., 2017).

### Online Monitor of pH Values in Algal Cultures

A total of three sets of customized transmitting systems and transmitters (BTE/PHG-96FS) were used to monitor pH values in algal cultures online. These systems were equipped with pH electrodes (InPro 3030, METTLER TOLEDO), and the accuracy was 0.1. The pH values in three algal cultures were recorded by a paperless recorder every 10 min, and the online monitor lasted for 48 h.

### Analyses of Biochemical Components

The biochemical components, including the total protein, carbohydrate, chlorophyll, and fatty acid profile, were determined by modified methods of Coomassie brilliant blue G250 colorimetry (Bradford, 1976), anthrone-H_2_SO_4_ colorimetry (Klein and Betz, 1978), ethanol colorimetry (Jespersen and Christoffersen, 1987), and one-step acid-catalyzed direct transesterification (Liu et al., 2015), respectively. The measurements were taken using the lyophilized biomass of *C*. *reinhardtii* BAFJ5 cells.

### Quantification of Glycerolipid Acyls Using Thin-Layer Chromatography and Gas Chromatograph

The lipid extraction, Thin-Layer Chromatography (TLC) separation of glycerolipids, and quantification of fatty acyls attaching to the individual glycerolipid class were performed using lyophilized *C. reinhardtii* BAFJ5 cells as previously described (Yang et al., 2018, 2020). The total lipids were extracted using chloroform/methanol/water (1:1:0.9, v/v/v) for three times, and then, the merged extracts were dried under gentle nitrogen flow. The re-dissolved lipids in chloroform were further separated into polar lipids and neutral lipids by TLC. The polar lipids included monogalactosyldiacylglycerol (MGDG), DGDG, sulphoquinovosyldiacylglycerol (SQDG), DGTS, phosphatidylethanolamine (PE), phosphatidylglycerol (PG), and phosphatidylinositol (PI). The neutral lipids were mainly composed of TAG. The polar lipids were developed in a mixture of chloroform/methanol/acetic acid/distilled water (75:13:9:3, v/v/v/v) and the neutral lipids in a mixture of hexane/diethyl ether/acetic acid (85:15:1, v/v/v) on TLC plates (TLC silica gel 60 F254; Merck KGA, Darmstadt, Germany). The separated lipids were then revealed through spraying with 0.05% (m/v) primuline (Sigma-Aldrich, St. Louis, United States) in acetone/water (80/20, v/v) at 365 nm. The silica-containing polar lipid class or TAG was scraped off and then esterified into fatty acid methyl esters followed by Gas Chromatograph (GC) quantification.

### Statistical Analysis

All the data were statistically analyzed using SPSS 19.0 and indicated as average ± standard deviation (*n* = 3). The statistical significance of the difference was assessed by one-way ANOVA Tukey’s HSD test and Student’s *t*-test denoted as *p < 0.05 or **p < 0.01.

## Results

### Changes in Growth, Photosynthetic Activity, and Biochemical Components in *Chlamydomonas reinhardtii* BAFJ5 Under Three Carbon Supply Regimes

**FIGURE 1:**
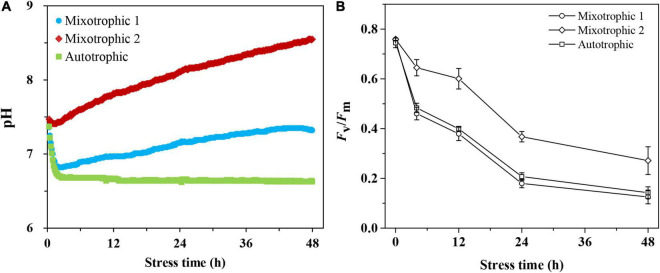
Time course of changes in pH **(A)** and photosynthetic activity **(B)** of *Chlamydomonas reinhardtii* BAFJ5 cultures under three carbon supply regimes over 48 h of HL-N. The carbon supply regimes for mixotrophic 1, mixotrophic 2, and autotrophic were 17 mM acetate and 2% CO_2_ (v/v), 17 mM acetate and ambient 0.04% CO_2_ (v/v), and 2% CO_2_ (v/v), respectively. The pH values of three algal cultures in A were recorded every 10 min, and data were derived from one batch culture. Values in B are the means of three independent replicates ± SD (*n* = 3). *F*_*v*_/*F*_*m*_ indicates the maximal quantum conversion efficiency.

The customized transmitting systems were used to monitor pH values in BAFJ5 cultures online under three carbon supply forms (Figure 1A). The pH values varied differently as the stress time was prolonged. The initial pH value, i.e., 7.4, rapidly decreased by 7 and 8% within 30 min under mixotrophic 1 and autotrophic conditions in BAFJ5 cultures, respectively, whereas the pH values remained constant in BAFJ5 cultures under mixotrophic 2 condition. After that, the pH value in BAFJ5 cultures under mixotrophic 2 condition increased by 16%, that under autotrophic condition decreased by 11%, and that under mixotrophic 1 condition recovered to 7.3 at 48 h of HL-N.

**FIGURE 2:**
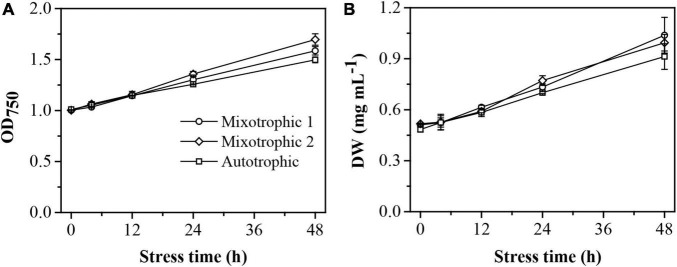
Time course of changes in growth performance of *C. reinhardtii* BAFJ5 cultures under three carbon supply regimes over 48 h of HL-N. The carbon supply regimes for mixotrophic 1, mixotrophic 2, and autotrophic were 17 mM acetate and 2% CO_2_ (v/v), 17 mM acetate and ambient 0.04% CO_2_ (v/v), and 2% CO_2_ (v/v), respectively. Values are the means of three independent replicates ± SD (*n* = 3). OD_750_, optical density; DW, dry weight density.

The maximum photosystem II (PS II) quantum conversion efficiencies, *F*_*v*_/*F*_*m*_, of the BAFJ5 cells were declined by 83, 64, and 81%, respectively, under mixotrophic 1, mixotrophic 2, and autotrophic conditions over 48 h of HL-N (Figure 1B). The BAFJ5 cells grown under mixotrophic 2 condition were found to be significantly higher in the maximum quantum conversion efficiencies (*F*_*v*_/*F*_*m*_) than those grown under mixotrophic 1 and autotrophic conditions, whereas there were no prominent differences between the cells grown under the latter two conditions. The growth parameter, optical density (OD_750_, Figure 2A), and dry weight density (DW, Figure 2B) all increased by 2-folds in *C*. *reinhadtii* cultures under three carbon supply regimes over 48 h of HL-N.

**FIGURE 3:**
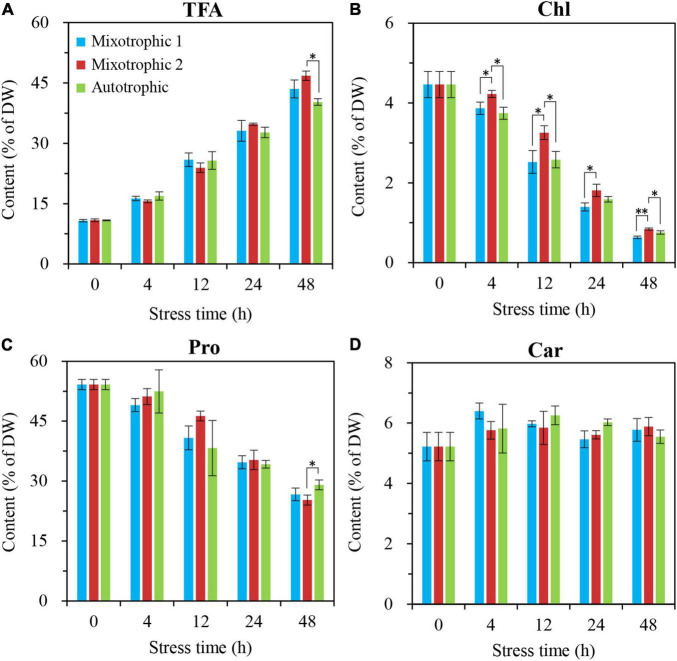
Time course of changes in amounts of total fatty acids **(A)**, chlorophyll **(B)**, protein **(C)**, and carbohydrate **(D)** in *C. reinhardtii* BAFJ5 under three carbon supply regimes over 48 h of HL-N. The carbon supply regimes for mixotrophic 1, mixotrophic 2, and autotrophic were 17 mM acetate and 2% CO_2_ (v/v), 17 mM acetate and ambient 0.04% CO_2_ (v/v), and 2% CO_2_ (v/v), respectively. Values are the means of three independent replicates ± SD (*n* = 3). TFAs, total fatty acids; Chl, chlorophyll; Pro, protein; Car, carbohydrate; DW, cellular dry weight; *p < 0.05; **p < 0.01.

The time course of alterations in biochemical constituents, including total fatty acid, Figure 3A, chlorophyll, Figure 3B, protein, Figure 3C, and carbohydrate, Figure 3D, was quantitatively analyzed to assess the physiological response of *C. reinhardtii* BAFJ5 cultured under the three trophic conditions to HL-N. The contents of total fatty acids in BAFJ5 cells under the three trophic conditions all rose by 4-folds in response to 48 h of HL-N. The chlorophyll contents of the algal cells grown under mixotrophic 1, mixotrophic 2, and autotrophic conditions were reduced by 86, 81, and 83%, respectively; the protein contents were reduced by 51, 53, and 46%, respectively. In particular, there were notably higher levels of chlorophyll in the BAFJ5 cells grown under the mixotrophic 2 condition than those grown under the other two trophic conditions during 48 h of HL-N, which was consistent with the case for PS II quantum conversion efficiency.

### Changes in the Individual Glycerolipid Class in *Chlamydomonas reinhardtii* BAFJ5 Under Three Carbon Supply Regimes

**FIGURE 4:**
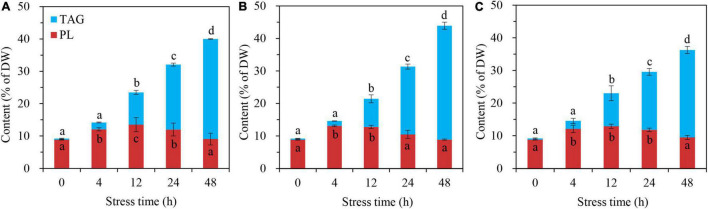
Time course of changes in contents of the total polar lipids and TAG in *C. reinhardtii* BAFJ5 under three carbon supply regimes over 48 h of HL-N. Panels **(A–C)** indicate mixotrophic 1 (17 mM acetate and 2% CO_2_, v/v), mixotrophic 2 (17 mM acetate and ambient 0.04% CO_2_, v/v), and autotrophic (2% CO_2_, v/v) conditions, respectively. PL indicates polar lipids, including monogalactosyldiacylglycerol (MGDG), digalactosyldiacylglycerol (DGDG), sulphoquinovosyldiacylglycerol (SQDG), diacylglycerol-*N*,*N*,*N*-trimethylhomoserine (DGTS), phosphatidylethanolamine (PE), phosphatidylglycerol (PG), and phosphatidylinositol (PI). Values are the means of three independent replicates (*n* = 3). The distinct letters labeled for the same parameter indicate the statistically significant difference by Tukey’s honestly significant difference (HSD) test. DW, cellular dry weight.

The glycerolipid responses of *C. reinhardtii* BAFJ5 to HL-N were compared under three tropical conditions. The BAFJ5 cells cultured under the three carbon supply regimes successively accumulated TAG as the stress time extended, and the contents of the total polar lipids increased first and decreased subsequently (Figures 4A–C).

**FIGURE 5:**
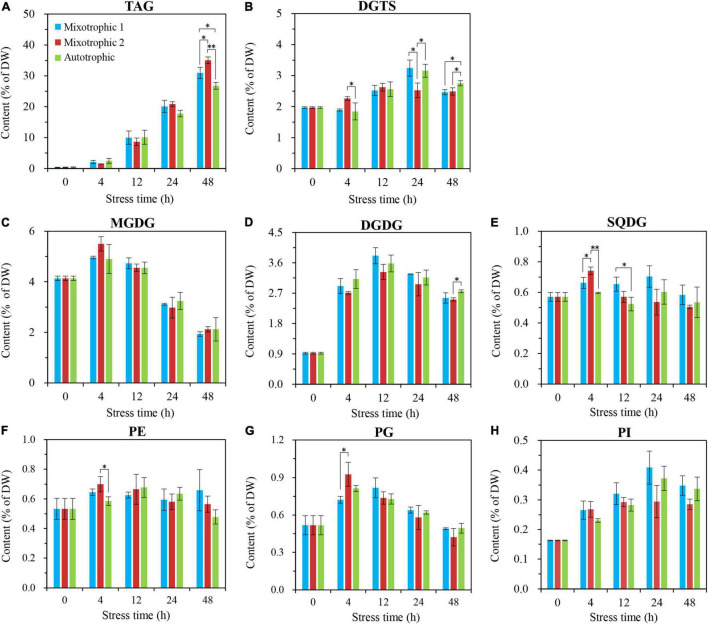
Time course of changes in amounts of the individual glycerolipid class in *C. reinhardtii* BAFJ5 under three carbon supply regimes over 48 h of HL-N. The carbon supply regimes for mixotrophic 1, mixotrophic 2, and autotrophic were 17 mM acetate and 2% CO_2_ (v/v), 17 mM acetate and ambient 0.04% CO_2_ (v/v), and 2% CO_2_ (v/v), respectively. Values are the means of three independent replicates ± SD (*n* = 3). DW, cellular dry weight; *p < 0.05; **p < 0.01.

No significant differences occurred in TAG contents of BAFJ5 cells among the three carbon supply regimes until 48 h of HL-N treatment, ranging as mixotrophic 2 > mixotrophic 1 > autotrophic (Figure 5A). The TAG amount in BAFJ5 cells grown under the mixotrophic 2 condition was shown to be 13 and 31% higher than those grown under mixotrophic 1 and autotrophic conditions, respectively. In addition, the levels of the major glycerolipids, including MGDG (Figure 5C), DGDG (Figure 5D), DGTS (Figure 5B), and the phospholipid PG as well as PI (Figures 5G,H), all showed an initial increase and a subsequent decrease trend, whereas that of other polar lipids, including SQDG and PE (Figures 5E,F), maintained relatively stable under HL-N conditions. Almost no prominent differences existed in the individual polar lipid amount among the algal cells cultured under the three carbon supply regimes.

### Changes in the Major Fatty Acid Profile of the Primary Glycerolipids in *Chlamydomonas reinhardtii* BAFJ5 Under Three Carbon Supply Regimes

To further investigate the cause of enhanced TAG accumulation in the BAFJ5 cells grown under mixotrophic 2 condition at 48 h of HL-N, the changes in the relative abundances of the major fatty acids in the primary glycerolipids were further monitored and analyzed.

**FIGURE 6:**
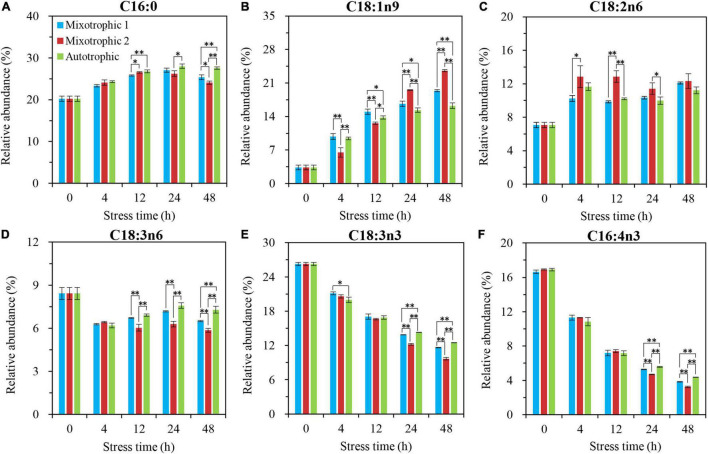
Time course of changes in the relative abundances of the major fatty acyls in *C. reinhardtii* BAFJ5 under three carbon supply regimes over 48 h of HL-N. The carbon supply regimes for mixotrophic 1, mixotrophic 2, and autotrophic were 17 mM acetate and 2% CO_2_ (v/v), 17 mM acetate and ambient 0.04% CO_2_ (v/v), and 2% CO_2_ (v/v), respectively. Cx:ynz, where x, y, and z refer to the number of carbon, the number of double bonds, and the position of the first double bond counting from the methyl end, respectively. Values are the means of three independent replicates ± SD (*n* = 3). *p < 0.05; **p < 0.01.

The most significantly different fatty acyls in the starchless BFAJ5 cultured under the three trophic conditions over 48 h of HL-N were found to be C18:1n9 (Cx:ynz, where x, y, and z refer to the number of carbon, the number of double bonds, and the position of the first double bond counting from the methyl end, respectively), and the remarkable difference occurred at 4 h (Figure 6). The BFAJ5 cells grown under the mixotrophic 2 condition first presented the most prominently lower abundances of C18:1n9 at 4 and 12 h, whereas the case was on the contrary at 24 and 48 h (Figure 6B). The relative levels of the polyunsaturated fatty acids, i.e., C18:3n6, C18:3n3, and C16:4n3 (Figures 6D–F), differed notably just at 24 and 48 h among the three trophically grown BFAJ5 cells. There were no drastic differences in the relative abundances of the saturated fatty acid C16:0 (Figure 6A) among the BFAJ5 cells under the different trophic modes until 48 h of HL-N.

**FIGURE 7:**
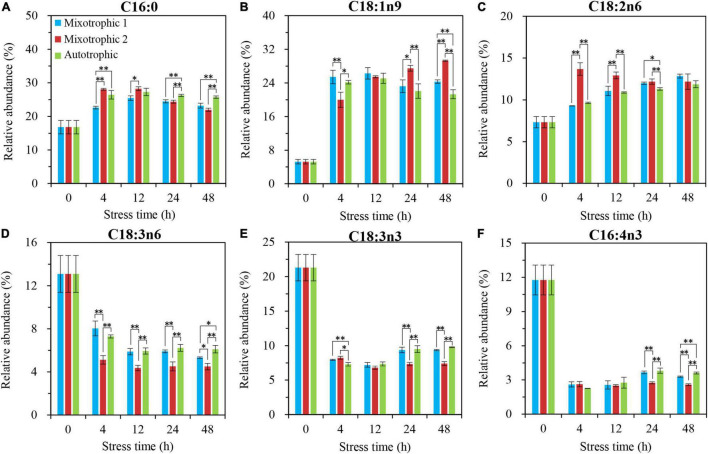
Time course of changes in the relative abundances of the major fatty acyls in TAG of *C. reinhardtii* BAFJ5 under three carbon supply regimes over 48 h of HL-N. The carbon supply regimes for mixotrophic 1, mixotrophic 2, and autotrophic were 17 mM acetate and 2% CO_2_ (v/v), 17 mM acetate and ambient 0.04% CO_2_ (v/v), and 2% CO_2_ (v/v), respectively. Cx:ynz, where x, y, and z refer to the number of carbon, the number of double bonds, and the position of the first double bond counting from the methyl end, respectively. Values are the means of three independent replicates ± SD (*n* = 3). *p < 0.05; **p < 0.01.

The relative abundances of C16:4n3 in TAG of *C. reinhardtii* BAFJ5 under three carbon supply regimes began to be significantly different at 24 h of HL-N (Figure 7F), whereas that of other five fatty acids, C16:0, C18:1n9, C18:2n6, C18:3n6, and C18:3n3, exhibited notable distinctions just at 4 h of HL-N (Figures 7A–E). The changes in the relative levels of the main fatty acids in TAG and the total fatty acids were similar in the three trophically grown BAFJ5 cells.

**FIGURE 8:**
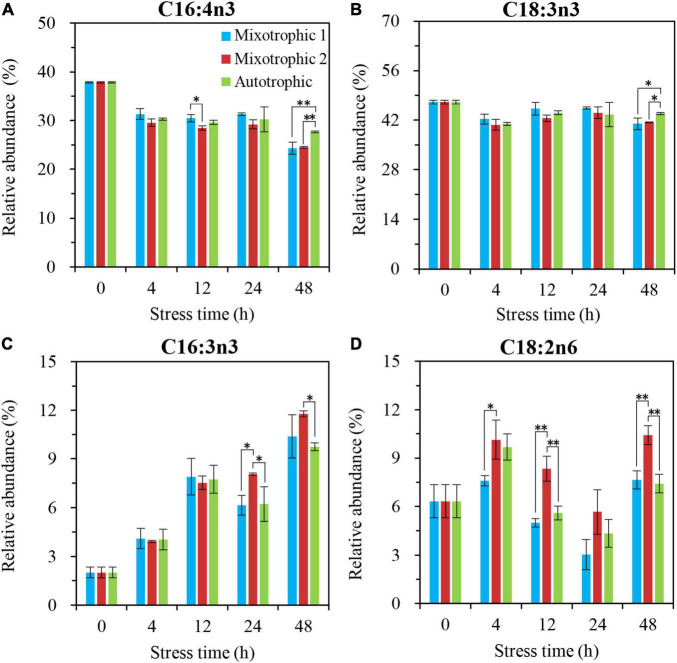
Time course of changes in the relative abundances of the major fatty acyls in MGDG of *C. reinhardtii* BAFJ5 under three carbon supply regimes over 48 h of HL-N. The carbon supply regimes for mixotrophic 1, mixotrophic 2, and autotrophic were 17 mM acetate and 2% CO_2_ (v/v), 17 mM acetate and ambient 0.04% CO_2_ (v/v), and 2% CO_2_ (v/v), respectively. Cx:ynz, where x, y, and z refer to the number of carbon, the number of double bonds, and the position of the first double bond counting from the methyl end, respectively. Values are the means of three independent replicates ± SD (*n* = 3). *p < 0.05; **p < 0.01.

The main fatty acids, C16:4n3 and C18:3n3, in MGDG of *C*. *reinhadtii* BAFJ5 grown under three trophic conditions did not show significant differences until 48 h of HL-N (Figures 8A,B). Their relative abundances in BAFJ5 cells grown autotrophically were notably higher than that grown mixotrophically. Another fatty acid, C16:3n3 (Figure 8C), showed prominent distinction at 24 h of HL-N among the three trophic conditions, and its relative levels for cells grown under the mixotrophic 2 condition were remarkably higher than those under the autotrophic condition. Differently, the fatty acid, C18:2n6, started to present prominent differences at 4 h of HL-N (Figure 8D). At 12 and 48 h of HL-N, the relative abundances of this fatty acid in cells grown under the mixotrophic 2 condition were obviously higher than those grown under mixotrophic 1 and autotrophic conditions.

**FIGURE 9:**
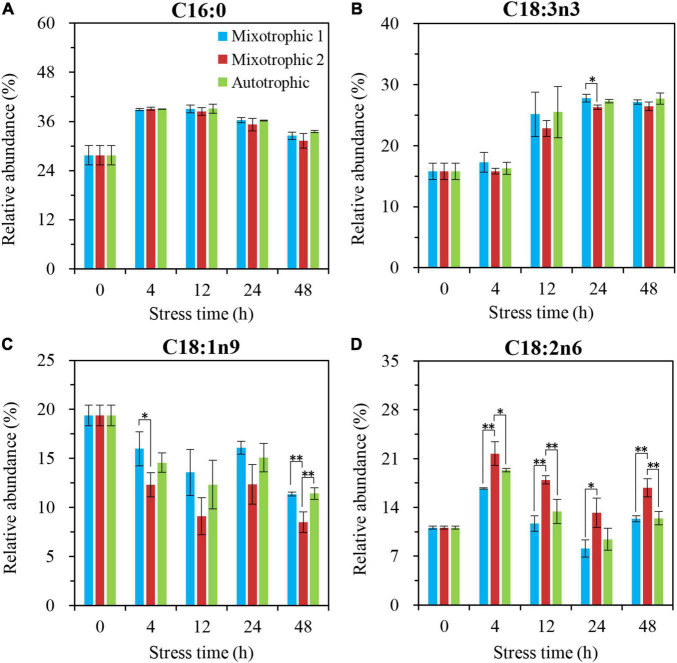
Time course of changes in the relative abundances of the major fatty acyls in DGDG of *C. reinhardtii* BAFJ5 under three carbon supply regimes over 48 h of HL-N. The carbon supply regimes for mixotrophic 1, mixotrophic 2, and autotrophic were 17 mM acetate and 2% CO_2_ (v/v), 17 mM acetate and ambient 0.04% CO_2_ (v/v), and 2% CO_2_ (v/v), respectively. Cx:ynz, where x, y, and z refer to the number of carbon, the number of double bonds, and the position of the first double bond counting from the methyl end, respectively. Values are the means of three independent replicates ± SD (*n* = 3). *p < 0.05; **p < 0.01.

The primary fatty acids, C16:0 and C18:3n3, in DGDG of *C*. *reinhardtii* BAFJ5 cells cultured under three carbon supply regimes showed similar variation in the relative levels over 48 h of HL-N (Figures 9A, B). The BAFJ5 cells grown under the mixotrophic 2 condition had notably higher relative levels of C18:1n9 in DGDG than those grown under autotrophic and mixotrophic 1 conditions at 48 h of HL-N (Figure 9C). Distinctly, the relative abundances of C18:2n6 in DGDG of BAFJ5 cells under the mixotrophic 2 condition were prominently higher than the other two conditions just from 4 h of HL-N (Figure 9D).

**FIGURE 10:**
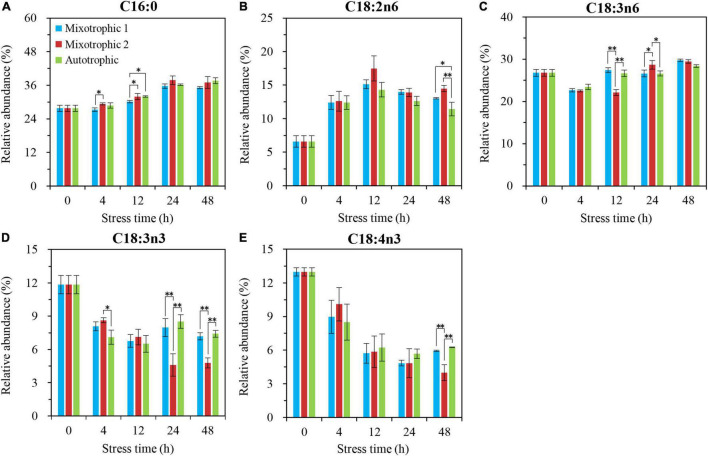
Time course of changes in the relative abundances of the major fatty acyls in DGTS of *C. reinhardtii* BAFJ5 under three carbon supply regimes over 48 h of HL-N. The carbon supply regimes for mixotrophic 1, mixotrophic 2, and autotrophic were 17 mM acetate and 2% CO_2_ (v/v), 17 mM acetate and ambient 0.04% CO_2_ (v/v), and 2% CO_2_ (v/v), respectively. Cx:ynz, where x, y, and z refer to the number of carbon, the number of double bonds, and the position of the first double bond counting from the methyl end, respectively. Values are the means of three independent replicates ± SD (*n* = 3). *p < 0.05; **p < 0.01.

In contrast to MGDG and DGDG, the relative abundances of the major fatty acids in DGTS showed minor differences between BAFJ5 cells under three carbon supply regimes (Figure 10). Among that, the BAFJ5 cells grown under the mixotrophic 2 condition had significantly lower levels of C18:3n3 than those under the other two conditions since 24 h of HL-N (Figure 10D). In addition, the case for C18:4n3 (Figure 10E) was similar to C18:3n3, and that for C18:2n6 (Figure 10B) was converse at 48 h of HL-N.

### Changes in the Glycerolipid Profiles in *Chlamydomonas reinhardtii* BAFJ5 Under Three Carbon Supply Regimes

**FIGURE 11:**
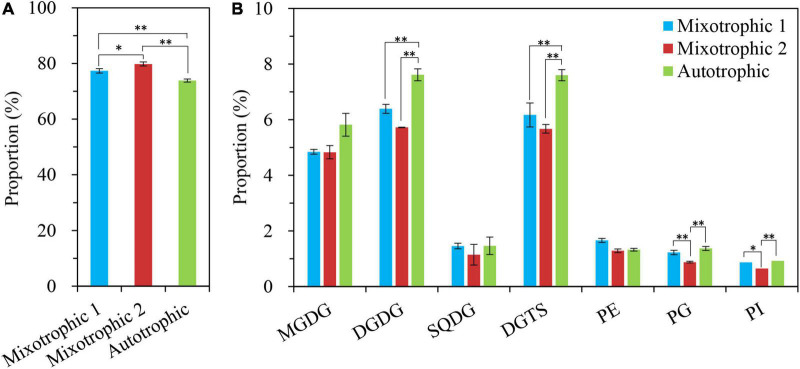
Proportions (%) of the glycerolipid components in *C. reinhardtii* BAFJ5 under three carbon supply regimes at 48 h of HL-N. A: TAG; B: polar lipids. The carbon supply regimes for mixotrophic 1, mixotrophic 2, and autotrophic were 17 mM acetate and 2% CO_2_ (v/v), 17 mM acetate and ambient 0.04% CO_2_ (v/v), and 2% CO_2_ (v/v), respectively. GL, glycerolipid; TAG, triacylglycerol; MGDG, monogalactosyldiacylglycerol; DGDG, digalactosyldiacylglycerol; SQDG, sulphoquinovosyldiacylglycerol; DGTS, diacylglycerol-*N*,*N*,*N*-trimethylhomoserine; PE, phosphatidylethanolamine; PG, phosphatidylglycerol; and PI, phosphatidylinositol. Values are means of three independent replicates ± SD (*n* = 3). *p < 0.05; **p < 0.01.

The changes in proportions of TAG and the individual polar lipid class in the total glycerolipids were compared over 48 h of HL-N (Table 1). The percentage of TAG in total lipids in starchless BAFJ5 under three trophic modes gradually increased by 20-folds as the stress time prolonged. At 24 h, the proportion of TAG in BAFJ5 cells grown under the mixotrophic 2 condition was significantly higher than those under autotrophic and mixotrophic 1 conditions. Up to 48 h, the prominent differences occurred in the relative levels of TAG between the latter two conditions (Figure 11A).

**TABLE 1:** Time course of changes in the glycerolipid profiles (%) of *Chlamydomonas reinhardtii* BAFJ5 under three carbon supply regimes over 48 h of HL-N.

GL	0*h*	4 h			12 h			24 h			48 h		
		Mixotrophic 1	Mixotrophic 2	Autotrophic	Mixotrophic 1	Mixotrophic 2	Autotrophic	Mixotrophic 1	Mixotrophic 2	Autotrophic	Mixotrophic 1	Mixotrophic 2	Autotrophic
TAG	3.9 ± 0.9	14.7 ± 2.9	10.2 ± 0.3	16.6 ± 3.5	42.2 ± 4.6	40.3 ± 3.4	43.5 ± 4.7	62.6 ± 1.5*^b^*	66.7 ± 1.9^a^	60.1 ± 1.4^b^	77.4 ± 0.8^b^	79.8 ± 0.7^a^	73.9 ± 0.6^c^
MGDG	45.1 ± 0.1	35.1 ± 1.3^bc^	37.7 ± 1.3^ab^	33.7 ± 0.6^c^	20.3 ± 1.6	21.3 ± 1.7	19.9 ± 1.8	9.7 ± 0.7	9.5 ± 0.7	10.9 ± 0.8	4.8 ± 0.3	4.8 ± 0.2	5.8 ± 1.0
DGDG	10.1 ± 0.5	20.5 ± 0.7^a^	18.6 ± 0.1^b^	21.5 ± 1.0^a^	16.3 ± 1.0	15.5 ± 0.3	15.6 ± 1.0	10.2 ± 0.8	9.4 ± 0.5	10.7 ± 1.2	6.4 ± 0.1^b^	5.7 ± 0.2^b^	7.6 ± 0.4^a^
SQDG	6.2 ± 0.2	4.7 ± 0.5	5.1 ± 0.2	4.1 ± 0.6	2.8 ± 0.3^ab^	2.7 ± 0.2^bc^	2.3 ± 0.2^c^	2.2 ± 0.1^ab^	1.7 ± 0.2^c^	2.0 ± 0.2^bc^	1.5 ± 0.2	1.1 ± 0.0	1.5 ± 0.2
DGTS	21.5 ± 0.4	13.4 ± 0.7^b^	15.5 ± 0.4^a^	12.7 ± 0.2^b^	10.8 ± 0.6^c^	12.3 ± 0.3^ab^	11.2 ± 0.4^bc^	10.1 ± 0.1^a^	8.0 ± 0.3^b^	10.7 ± 0.2^a^	6.2 ± 0.1^b^	5.7 ± 0.4^b^	7.6 ± 0.3^a^
PE	5.8 ± 0.7	4.6 ± 0.4	4.8 ± 0.3	4.1 ± 0.7	2.7 ± 0.3	3.1 ± 0.6	3.0 ± 0.7	1.9 ± 0.1	1.9 ± 0.1	2.2 ± 0.2	1.7 ± 0.4	1.3 ± 0.2	1.3 ± 0.2
PG	5.7 ± 0.9	5.1 ± 0.4	6.4 ± 0.8	5.6 ± 0.7	3.5 ± 0.8	3.5 ± 0.4	3.2 ± 0.6	2.0 ± 0.2	1.8 ± 0.2	2.1 ± 0.1	1.2 ± 0.1^a^	0.9 ± 0.1^b^	1.4 ± 0.1^a^
PI	1.8 ± 0.1	1.9 ± 0.3	1.8 ± 0.2	1.6 ± 0.2	1.4 ± 0.1	1.4 ± 0.1	1.2 ± 0.1	1.3 ± 0.1^a^	0.9 ± 0.1^b^	1.3 ± 0.1^a^	0.9 ± 0.1^a^	0.6 ± 0.0^b^	0.9 ± 0.1^a^

*The carbon supply regimes for mixotrophic 1, mixotrophic 2, and autotrophic were 17 mM acetate and 2% CO_2_ (v/v), 17 mM acetate and ambient 0.04% CO_2_ (v/v), and 2% CO_2_ (v/v), respectively. Values are the means of three independent replicates ± SD (n = 3). Values (means of each time point) within the same line followed by the same lower-case letters differ with no significance (p > 0.05), and those followed by different lower-case letters differ significantly (p < 0.05). GL, glycerolipid; TAG, triacylglycerol; MGDG, monogalactosyldiacylglycerol; DGDG, digalactosyldiacylglycerol; SQDG, sulphoquinovosyldiacylglycerol; DGTS, diacylglycerol-N,N,N-trimethylhomoserine; PE, phosphatidylethanolamine; PG, phosphatidylglycerol; and PI, phosphatidylinositol. Values (means of each time point) within the same line followed by the same lower-case letters differ with no significance (P > 0.05), and those followed by different lower-case letters differ significantly (P < 0.05).*

The respective proportion of the major three polar lipids, i.e., MGDG, DGDG, and DGTS, in total lipids was prominently different among the three trophic modes at 4 h of HL-N. The BAFJ5 cells grown under the mixotrophic 2 condition had significantly higher proportions of MGDG and DGTS and a notably lower abundance of DGDG. The relative abundances of MGDG and DGTS in total lipids of BFAJ5 cells gradually decreased by more than 85 and 65% over 48 h of stress under the three carbon supply regimes, respectively. Importantly, the significantly different distinctions among the three trophic modes existed in DGTS throughout the whole period of HL-N. The relative levels of DGDG increased to the highest, i.e., 2-folds of the original level, at 4 h, and then decreased to the lowest, i.e., 57–76% of the original level, at 48 h (Figure 11B).

In addition, the other polar lipids, SQDG, PE, PG, and PI, in BAFJ5 cells all presented low distribution ratios, below 10%, in total lipids over 48 h of HL-N, and their distribution ratios were gradually decreased as the stress time prolonged. There were no remarkable differences for PE proportions over 48 h of stress. The obvious differences for proportions of PI and PG emerged until 24 and 48 h, respectively. The SQDG proportions were notably different at 12 and 24 h of HL-N.

## Discussion

Many studies were focused on TAG accumulation under distinct trophic cultivations using the wild-type algal cells, and the mixotrophically grown cells were found to produce more TAG than the photoautotrophically and heterotrophically grown cells (Shen et al., 2018, 2019). However, what is the case for the starchless mutant that redistributes its carbon flux and hyper-accumulates TAG? Current studies on the impacts of exogenous carbon on TAG accumulation are mostly performed in flasks. The inorganic carbon, i.e., CO_2_, is usually from the air, and 0.04% of CO_2_ appears to be insufficient to ensure algal cells’ complete growth and metabolism in relation to the supplied sufficient organic carbon, which inevitably covers up the authentic function of the inorganic carbon on TAG metabolism in algal cells. This study aims to make clear whether altering exogenous carbon supply, including organic and inorganic carbon, affected glycerolipid metabolism, TAG metabolism in particular, in starchless mutant BAFJ5 of *C*. *reinhardtii* under HL-N conditions.

### Similarity of Growth and Lipid Accumulation in Autotrophically and Mixotrophically Grown *Chlamydomonas reinhardtii* BAFJ5 With 2% CO_2_ as Inorganic Carbon Under HL-N Conditions

A previous study showed that the biomass in both the mixotrophically grown *C. vulgaris* and *S. obliquus* were 2-folds of that in the autotrophically grown cells (aerated with air containing 2% CO_2_) under nitrogen depletion with 2% CO_2_ as inorganic carbon source (Shen et al., 2018, 2019). In this study, the starchless *Chlamydomonas* BAFJ5 mutant defective in AGPase exhibited no difference in the growth of autotrophic and mixotrophic cultures aerated with air containing 2% CO_2_ during the whole period of HL-N stress.

Shen et al. (2018, 2019) reported that the fatty acid contents in nitrogen-depleted *C. vulgaris* and *S. obliquus* grown mixotrophically reached 62.6 and 44.1% of DW and were 3- and 2-folds of that grown photoautotrophically, respectively. It is worth noting that this difference also did not exist in HL-N-treated BAFJ5, and the fatty acid contents in BAFJ5 were both more than 40% of DW under photoautotrophic and mixotrophic conditions with 2% CO_2_ as inorganic carbon source (Figure 3A).

**FIGURE 12:**
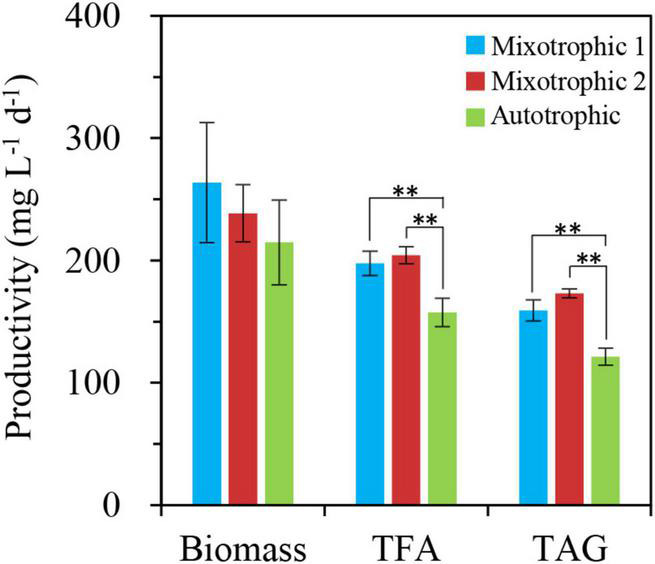
Productivities of biomass, total fatty acids, and TAG in *C. reinhardtii* BAFJ5 under three carbon supply regimes over 48 h of HL-N. The carbon supply regimes for mixotrophic 1, mixotrophic 2, and autotrophic were 17 mM acetate and 2% CO_2_ (v/v), 17 mM acetate and ambient 0.04% CO_2_ (v/v), and 2% CO_2_ (v/v), respectively. Values are the means of three independent replicates ± SD (*n* = 3). TFAs, total fatty acids. **p < 0.01.

In addition, the productivities of biomass and total fatty acids in 2% CO_2_-enriched mixotrophic cultures of *C. vulgaris* were demonstrated to be 4- and 14-folds of those in autotrophic cultures after 6 days of nitrogen starvation, respectively (Shen et al., 2018). Similarly, the corresponding distinctions between the autotrophic and mixotrophic cultures in *S. obliquus* also reached 2- and 7-folds, respectively (Shen et al., 2019). In this study, the mixotrophic 1 and autotrophic cultures presented no prominent differences in biomass productivity under HL-N conditions (Figure 12). Moreover, the productivities of total fatty acids and TAG in mixotrophically grown BAFJ5 cells with 2% CO_2_ as inorganic carbon source (mixotrophic 1) were 26 and 31% higher than that in autotrophically grown cells; the mixotrophically grown BAFJ5 with 0.04% CO_2_ as inorganic carbon source (mixotrophic 2) had more superior productivities, i.e., 30 and 43% higher, in total fatty acids and TAG than autotrophic cultures (Figure 12). It is likely that the starchless phenotype resulted in the differences in biomass and lipid productivities among the distinct trophic cultures much smaller.

The great difference in growth and lipid accumulation between the wild-type and starchless mutant of green algae revealed that the starchless phenotype indeed altered physiological metabolism in green algae, mainly the flow of carbon flux into storage compounds, e.g., lipid and starch. The starch and lipid biosynthesis are the two competing pathways for carbon storage, with starch biosynthesis dominating over lipid accumulation (Fan et al., 2012). Although the carbon flows into lipids were prominently different between the autotrophic and mixotrophic cultures, the carbon flows into starch were correspondingly different in nitrogen-depleted wild-type green algae. Once the starch biosynthesis was inhibited, the storage carbon was mostly shunted into lipid, regardless of the exogenous carbon supply form. In that case, the lipid responses of starchless BAFJ5 were thus slightly different from each other under the three carbon supply regimes in this study.

### Unique Triacylglycerol Metabolism in Mixotrophically Grown *Chlamydomonas reinhardtii* BAFJ5 With 0.04% CO_2_ as Inorganic Carbon Under HL-N Conditions

Li et al. (2010a) found that the TAG content in *C*. *reinhardtii* starchless mutant BAFJ5 presented 30% higher under nitrogen-free TAP cultures than that under nitrogen-free HS cultures (autotrophic condition). However, it might not truly reflect the distinction of TAG metabolism between mixotrophic and autotrophic cultivations. Because the two media contain different levels of nitrogen and phosphorus apart from the carbon source. In this study, the media in autotrophic and mixotrophic cultures were kept the same apart from the supplied carbon. Both the autotrophic and mixotrophic 1 cultures were aerated with air containing 2% CO_2_, though the mixotrophic 1 cultures were added with sufficient organic acetate. Our results revealed that the TFA content of algal cells under mixotrophic 1 and mixotrophic 2 conditions was 8 and 16% (calculated from data in Figure 3A) higher than that under autotrophic condition. More importantly, the TAG content of algal cells under the two mixotrophic conditions was 16 and 31% (calculated from data in Figure 5A) higher than that under autotrophic condition. Hence, acetate was ascertained again to increase TAG accumulation in *Chlamydomonas*, not only in wild type, but also in the starchless mutant, which was consistent with the previous study (Fan et al., 2012). In addition, there was no significant difference occurred in the lipid accumulation between the autotrophic and mixotrophic 1 cultures on the condition that the aerated CO_2_ was adequate. It was assumed that the starchless BAFJ5 assimilated the inorganic CO_2_ in priority to the acetate once the inorganic carbon source was abundant under nitrogen starvation conditions. At that time, the organic acetate was proposed to function negligibly on lipid accumulation, and the assimilation of acetate in mixotrophic BAFJ5 cultures still needs further confirmation in our future work. When the inorganic CO_2_ was utilized as the carbon source, it was initially fixed into glucose with the catalysis of Rubisco and a series of synthetic reactions. Then, the generated pyruvate through glycolysis pathways was further decarboxylated into acetyl-CoA, and the acetyl-CoA subsequently entered into the fatty acid biosynthesis pathways. These pathways consumed certain amounts of reducing equivalents and energy, serving as the necessities for the assembly of fatty acids (Smith and Gilmour, 2018). However, the mixotrophically grown cells with 0.04% CO_2_ as inorganic carbon (mixotrophic 2 condition) might directly utilize acetate in the culture medium to form fatty acids without additional carbon fixation and the correlated transformation pathways. Thus, the mixotrophic cultures with 0.04% CO_2_ as inorganic carbon yielded higher content (Figures 4B, 5A) as well as proportion (Figure 11A and Table 1) of storage TAG than the autotrophic and mixotrophic cultures with 2% CO_2_ as inorganic carbon. On the other hand, Juergens et al. (2016) found that, in mixotrophic culture similar to mixotrophic 2 in our study, the starch was mainly formed using CO_2_, and fatty acid biosynthesis was largely dependent on exogenous acetate in the wild type of *C. reinhardtii*, based on a 13C labeling time-course study. However, to our knowledge, the case for the starchless mutant on the preferential utilization of CO_2_ and acetate still remains unclear in stress-induced *C. reinhardtii*. This study provides potentially useful insights into the carbon utilization in starchless *C. reinhardtii*, to a certain extent, which still needs further exploration in our future work. In view of pH values, the mixotrophic cultures with 0.04% CO_2_ showed the highest pH values, i.e., up to 8.5 at 48 h of HL-N (Figure 1A), compared with the other two cultures. The alkaline pH likely poses stress to the BAFJ5 cells, resulting in more enhanced TAG accumulation. In this case, the pH stress might turn out to be another vital factor affecting TAG accumulation. More pH stress contributes more to TAG production, which was in line with the results in the previous study (Gardner et al., 2013).

Under heterotrophic culture conditions, the neutral lipid content was below 5% of DW in both the WT and BAFJ5 as the previous study reported (Li et al., 2010b). However, the mixotrophically grown cells with 0.04% CO_2_ as inorganic carbon accumulated TAG up to 35% of DW in this study, suggesting the essential roles of light in TAG accumulation. The limited accumulation of TAG in the dark is not solely due to the restricted acetate uptake, but rather may reflect the dependency of storage product synthesis on the supply of ATP and/or NADPH through light reactions of photosynthesis. The generated energy and reducing equivalent are subsequently utilized by anabolic reactions in the cells (Li-Beisson et al., 2019). Exogenous acetate is presumed to be transported into the cell *via* a monocarboxylate transporter. Intracellular acetate in multiple organelles could be transformed into acetyl-CoA by two alternative processes involving acetyl-CoA synthase (ACS) or the two-step reactions involving phosphate acetyltransferase (PAT) and acetate kinase (AK) (Smith and Gilmour, 2018). It was interesting to note that the high light damage extent of mixotrophically grown BAFJ5 cells with 0.04% CO_2_ as inorganic carbon turned out to be the least as shown by the photosynthetic activity (Figure 1B) and chlorophyll levels (Figure 3B) when exposed to HL-N conditions for 48 h. In this case, the released reductant NADPH and energy ATP were more effectively used for anabolic reactions, and the fatty acid synthesis precursor acetate was also sufficient to support the formation of storage TAG.

In view of biomass and fatty acid productivities, the starchless BAFJ5 exhibited superior advantages compared to the non-starchless industrial algal strains, such as *C. vulgaris* and *S. obliquus*, under nitrogen-starved conditions (Table 2). Although the mixotrophic cultures with 0.04 or 2% CO_2_ as inorganic carbon showed no prominent differences between the biomass and fatty acid productivities, especially the TAG productivity (Figure 12), the former needed no additional CO_2_ supplement with high cost. Hence, the mixotrophic cultivation of starchless green algae under HL-N condition with 0.04% CO_2_ as inorganic carbon turned out to be a potential effective strategy to produce the storage reserve TAG without compromised growth.

**TABLE 2:** Biomass and fatty acid productivities in nitrogen-deprived green algae under distinct carbon supply regimes.

Parameter	Microalgae	Mixotrophic 1	Mixotrophic 2	Heterotrophic	Photoautotrophic	References
Biomass productivity	*Chlorella vulgaris*	211.5 ± 37.6	−	83.5 ± 5.3	55.4 ± 1.7	Shen et al., 2019
	*Scenedesmus obliquus*	207.7 ± 4.8	−	92.3 ± 1.8	89.4 ± 4.2	Shen et al., 2018
	*Chlamydomonas reinhardtii* BAFJ5	263.7 ± 49.1	238.6 ± 23.3	−	214.8 ± 34.7	This study
Fatty acid productivity	*Chlorella vulgaris*	149.0 ± 24.0	−	72.6 ± 3.9	11.0 ± 0.9	Shen et al., 2019
	*Scenedesmus obliquus*	110.6 ± 5.2	−	55.9 ± 2.4	16.2 ± 1.7	Shen et al., 2018
	*Chlamydomonas reinhardtii* BAFJ5	197.7 ± 9.8	204.3 ± 7.1	−	157.4 ± 7.0	This study

*Mixotrophic 1 and mixotrophic 2 denote mixotrophic conditions with 0.04% and 2% CO_2_ as inorganic carbon sources, respectively.*

### Vital Function of Membrane Lipids in Triacylglycerol Accumulation in *Chlamydomonas reinhardtii* BAFJ5

At present, the lipidomic responses in stress-induced microalgae, commonly the wild-type cells, have been largely reported because of the increasing demands for renewable biofuels from microalgae (Fan et al., 2011; Martin et al., 2014; bida et al., 2015; Matich et al., 2018). The microalgal cultures in many studies were already at the late stage of stress induction, when the membrane lipids usually greatly degraded or remained constant. However, in this study, the starchless BAFJ5 first increased and then decreased the amounts of the major membrane lipids, MGDG, DGDG, and DGTS, under all the three carbon supply regimes as HL-N stress time extended (Figure 5). In particular, the great net accumulation of DGDG and DGTS was observed to be the most prominent, which was obviously different from the decline, invariability, or slight increase of polar lipids in wild-type cells (Yoon et al., 2012; Zang et al., 2020). In addition, the polar glycerolipids in *C*. *reinhardtii* BAFJ5 responded to the HL-N conditions differently in time course. It was found that MGDG and DGDG responded first within 4 h, whereas DGTS showed a delayed alteration until 12 h of HL-N. These characteristics were present in starchless BAFJ5 under autotrophic and mixotrophic HL-N conditions, and it once again showed that the polar lipids could be accumulated indeed following HL-N as our previous study reported (Yang et al., 2020). These findings highlighted the unique lipid metabolism in starchless mutants of green microalgae.

**FIGURE 13:**
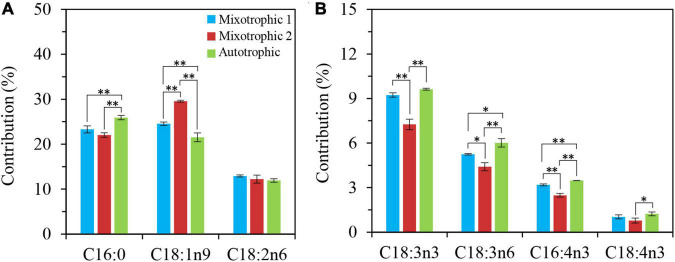
Contribution of certain fatty acid to TAG accumulation in *C. reinhardtii* BAFJ5 under three carbon supply regimes over 48 h of HL-N. Cx:ynz, where x, y, and z refer to the number of carbon, the number of double bonds, and the position of the first double bond counting from the methyl end, respectively. The contribution (%) is calculated as Con (%) = (M_t_ – M_0_)/(M_TAGt_ – M_TAG0_) × 100%, where Con (%) means the contribution of certain fatty acid in TAG to accumulation of TAG; M_t_ and M_0_ mean the content of certain fatty acid in TAG at the end and start of HL-N, respectively; M_TAGt_ and M_TAG0_ mean the content of TAG at the end and start of HL-N, respectively. *P < 0.05; **P < 0.01.

The fatty acid composition in membrane lipids could change under various abiotic stress conditions. The HL-N stress led to membrane lipid remodeling and the distinct carbon availability further enhanced this remodeling, i.e., the great alteration in fatty acid compositions in this study. In terms of time-course changes, the fatty acid compositions in membrane lipids, i.e., MGDG, DGDG, and DGTS, varied consistently with that in our previous study supplying 2% CO_2_ under mixotrophic conditions (Yang et al., 2020). In addition, the mixotrophically grown BAFJ5 cells with 0.04% CO_2_ as inorganic carbon source were found to be largely different from the mixotrophically and autotrophically grown cells with 2% CO_2_ as inorganic carbon source in the polar glycerolipid response. The BAFJ5 cells under 0.04% CO_2_-enriched mixotrophic mode increased the relative abundances of C18:2n6 in MGDG (Figure 8D) and DGDG (Figure 9D) in priority and decreased that of C18:3n3 and C18:4n3 in DGTS (Figure 10), subsequently, to respond to HL-N stress conditions. These particular responses in terms of membrane lipids probably contributed to the prominent accumulation of TAG in 0.04% CO_2_-enriched mixotrophic cultures over 48 h of HL-N. On the other hand, the monounsaturated C18:1n9 made the largest contribution to TAG accumulation in mixotrophically grown BAFJ5 with 0.04% CO_2_ as inorganic carbon source, which was dramatically higher than that in the other trophic cultures (Figure 13A). Conversely, the remarkably lower contributions of the polyunsaturated C18:3n3, C18:3n6, and C16:4n3, to TAG assembly (Figure 13B) were noticed in HL-N treated BAFJ5 under mixotrophic conditions with 0.04% CO_2_ as inorganic carbon source. It was demonstrated that the *de novo* biosynthesis pathway performed the primary function in the accumulation of saturated and monounsaturated TAG in mixotrophically grown BAFJ5 with 0.04% CO_2_ as an inorganic carbon source. The *de novo* synthesized C18:1n9 likely served as acyl-ACP or acyl-CoA pool to further be esterified into TAG within or outside the chloroplast. In addition, the accumulation of C18:1n9-attached TAG might be attributed to the degradation of the newly synthesized MGDG, which was probably catalyzed by the galactoglycerolipid lipase, PGD1 (Li et al., 2012). Moreover, the turnover pathways involving the membrane lipids, mainly MGDG, DGDG, and DGTS, were proposed to function slightly inferior for polyunsaturated TAG increment in 0.04% CO_2_-enriched mixotrophic cultures. It implied the complicated TAG assembly mechanisms presumably involving turnover of the nascent or newly-formed membrane lipids (Yang et al., 2020) under distinct carbon supply regimes, which still needed further exploitation in our future work. Our results suggested that the carbon supply regime serves as a pivotal factor to control TAG production involving the turnover of membrane lipids in starchless *Chlamydomonas*. These findings have broad implications not only for advancing understanding of modulation of TAG biosynthesis correlated with membrane lipid remodeling but also for physiological strategies to develop starchless green microalgae-based biofuel production systems.

## Conclusion

Based on the glycerolipid response of *C*. *reinhardtii* starchless BAFJ5 to three carbon supply regimes under HL-N conditions, it was revealed that the autotrophically and mixotrophically grown BAFJ5 cells with 2% CO_2_ as the inorganic carbon source presented similar changes in growth, photosynthetic activity, biochemical components, and glycerolipid metabolism under HL-N conditions. Distinctly, the mixtrophically grown *C*. *reinhardtii* BAFJ5 with 0.04% CO_2_ as inorganic carbon source exhibited a more superior accumulation of TAG; these TAGs had significantly higher levels of C18:1n9 and notably lower levels of polyunsaturated fatty acids. The BAFJ5 cells grown under 0.04% CO_2_-enriched mixotrophic condition increased the relative levels of C18:2n6 in membrane lipids, MGDG and DGDG, and decreased that of C18:3n3 and C18:4n3 in the betaine lipid, DGTS, to adapt to the HL-N stress. Thus, it was shown that the mixotrophy with 0.04% CO_2_ as inorganic carbon source endowed the starchless mutant of *C*. *reinhardtii* with peculiar glycerolipid response, including superior TAG accumulation and prominent polar lipid remodeling under HL-N conditions. This study offers useful insights into different impacts of different carbon supply strategies on lipid metabolism in *Chlamydomonas* starchless cells and will benefit the effective production of storage lipids in microalgae.

## Data Availability Statement

The original contributions presented in the study are included in the article/supplementary material, further inquiries can be directed to the corresponding authors.

## Author Contributions

MY, XX, SX, and ZG conceived and designed the research. MY and XX performed the experiments and wrote the manuscript. MY, XX, and SX analyzed the data and revised the manuscript. F-TK assisted in revision of the manuscript. K-PX, S-HY, J-YM, and ZG participated in preparation of the manuscript. SX and ZG supervised all the experiments. All authors agreed on the manuscript and approved the submitted version.

## Conflict of Interest

The authors declare that the research was conducted in the absence of any commercial or financial relationships that could be construed as a potential conflict of interest.

## Publisher’s Note

All claims expressed in this article are solely those of the authors and do not necessarily represent those of their affiliated organizations, or those of the publisher, the editors and the reviewers. Any product that may be evaluated in this article, or claim that may be made by its manufacturer, is not guaranteed or endorsed by the publisher.

## Footnotes

1
http://www.chlamycollection.org/
